# Target Therapies for Systemic Mastocytosis: An Update

**DOI:** 10.3390/ph15060738

**Published:** 2022-06-11

**Authors:** Mariarita Sciumè, Claudio De Magistris, Nicole Galli, Eleonora Ferretti, Giulia Milesi, Pasquale De Roberto, Sonia Fabris, Federica Irene Grifoni

**Affiliations:** 1 Hematology Unit, Fondazione IRCCS Ca’ Granda Ospedale Maggiore Policlinico, 20122 Milan, Italy; giulia.milesi@policlinico.mi.it (G.M.); pasquale.deroberto@policlinico.mi.it (P.D.R.); sonia.fabris@policlinico.mi.it (S.F.); federica.grifoni@policlinico.mi.it (F.I.G.); 2 Department of Oncology and Oncohaematology, Università degli Studi di Milano, 20122 Milan, Italy; claudio.demagistris@unimi.it (C.D.M.); nicole.galli@unimi.it (N.G.); 3 Direzione Scientifica, Fondazione IRCCS Ca’ Granda Ospedale Maggiore Policlinico, 20122 Milan, Italy; eleonora.ferretti@policlinico.mi.it

**Keywords:** systemic mastocytosis, target therapy, midostaurin, imatinib, avapritinib

## Abstract

Systemic mastocytosis (SM) results from a clonal proliferation of abnormal mast cells (MCs) in extra-cutaneous organs. It could be divided into indolent SM, smoldering SM, SM with an associated hematologic (non-MC lineage) neoplasm, aggressive SM, and mast cell leukemia. SM is generally associated with the presence of a gain-of-function somatic mutation in *KIT* at codon 816. Clinical features could be related to MC mediator release or to uncontrolled infiltration of MCs in different organs. Whereas indolent forms have a near-normal life expectancy, advanced diseases have a poor prognosis with short survival times. Indolent forms should be considered for symptom-directed therapy, while cytoreductive therapy represents the first-line treatment for advanced diseases. Since the emergence of tyrosine kinase inhibitors (TKIs), *KIT* inhibition has been an attractive approach. Initial reports showed that only the rare KITD816V negative cases were responsive to first-line TKI imatinib. The development of new TKIs with activity against the KITD816V mutation, such as midostaurin or avapritinib, has changed the management of this disease. This review aims to focus on the available clinical data of therapies for SM and provide insights into possible future therapeutic targets.

## 1. Introduction

Systemic mastocytosis (SM) is a WHO-defined hematological malignancy, which now has its nosological independence from myeloproliferative neoplasms and is characterized by clonal proliferation of mast cells (MCs) in the bone marrow (BM) and/or in organs other than skin [1,2]. 

SM encompasses different clinicopathological subgroups, differing in clinical course, laboratory findings and histological features [1,2].

More than 90% of SM patients harbor a somatic gain-of-function mutation in the *KIT* gene, namely KITD816V, that leads to a constitutional activity of the *KIT* receptor in a ligand-independent fashion [3]. It has been demonstrated that SM cases with multilineage *KIT* mutation cluster more frequently with advanced mastocytosis (AdvSM) than indolent cases [4].

A diagnosis of SM is made if one major criterion (BM multifocal or dense cluster of ≥15 atypical MCs) and one minor criterion are met or, alternatively, if three minor criteria are fulfilled. Minor criteria are represented by: the presence of ≥25% atypical or spindle-shaped MCs in the BM or extracutaneous organ infiltrates; detection of a KITD816V point mutation in the blood, BM or other extracutaneous organs; CD2 and/or CD25 expression on MCs surface; elevation of serum tryptase exceeding 20 ng/mL (unless there is an associated myeloid neoplasm, in which case this parameter is not valid) [1,2]. 

This review aims to focus on the available clinical data of therapies for SM and provide insights into possible future therapy targets; underlying mechanisms are explained and an overview of clinical experience with phase I–III studies is given.

## 2. Pathogenesis and Molecular Aspects of Systemic Mastocytosis

MCs develop from hematopoietic progenitor cells in the BM. After a partial differentiation, they are released as precursors in the bloodstream, reach tissues and organs, where complete maturation occurs, and acquire a tissue-specific phenotype. The CD117 transmembrane tyrosine kinase receptor, encoded by KIT, mainly regulates the growth, migration, survival, and effector functions of MCs [5].

The contributions of MCs to allergic and other inflammatory reactions are now well established and MCs are recognized as key effector cells in IgE-dependent allergic inflammation [5,6,7]. 

MCs express high-affinity receptors for IgE and produce numerous biologically active substances, some of which are stored in cytoplasmic granules for rapid release [6]. Many different agents may play the role of MC activators. These agents include allergens that can act through allergen-specific IgE and IgE receptors, cytokines, anaphylatoxins, neuropeptides, physical stimuli (pressure and temperature changes), exogenous toxins, and IgG immune complexes and complement, drugs, and bacterial and viral products [5,6,7]. 

Activation of MCs leads to degranulation and secretion of numerous vasoactive and pro-inflammatory mediators that contribute to the multiple symptoms observed in patients. Preformed molecules stored in secretory granules include histamine, serotonin, proteases (e.g., tryptase, chymase, and carboxypeptidase), and tumor necrosis factor (TNF). MCs secrete mitochondrial DNA, which carries out autocrine and paracrine stimulatory actions [5,6,7,8]. 

In addition, MCs release newly synthesized leukotrienes, prostaglandins, and platelet-activating factors, as well as many cytokines (including interleukin-6, interleukin-9, interleukin-13, and TNF) and chemokines (CXCL8, CCL2, and CCL5) [5,6,7]. 

**Figure 1:**
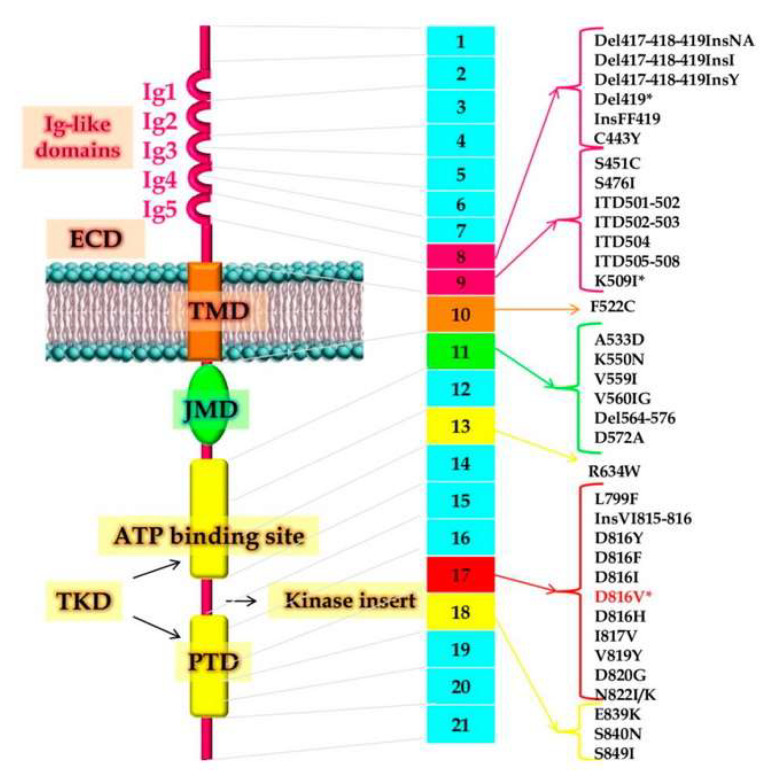
The image shows the structure of the KIT receptor with the known function of its domains and localization of all reported KIT mutations in adult patients with mastocytosis. On the left the structure of the receptor; in the center, the 21 KIT exons and the most frequently reported mutations. *: mutation found in >80% of adult patients with systemic mastocytosis. Abbreviations: Del, deletion; ECD, extracellular domain; Ins, insertion; ITD, internal tandem duplication; JMD, juxtamembrane domain; TKD, tyrosine kinase domain; PTD, phosphotransferase domain; TMD, transmembrane domain. Image source Ref. [9].

In SM, the proliferation of abnormal clonal MCs could interest different cutaneous and extracutaneous organs [1,2]. The molecular mechanisms that underlie SM development are currently not well-understood. Over 80% of patients with SM harbor the gain-of-function mutation in codon 816 of the tyrosine kinase KIT, where valine is substituted for an aspartate (KITD816V mutation). Other less common (<5%) somatic *KIT* mutations identified in adult SM are V560G, D815K, D816Y, insVI815-816, D816F, D816H, and D820G (Figure 1) [2,3,4,9]. However, *KIT* mutations do not occur universally, and the question if individual mutations are sufficient to generate MC transformation and to explain alone the different clinical presentations of SM remains unsettled. *KIT* appears to be a weak oncogene and could represent a late event in the pathogenesis of mastocytosis [2,3,4]. 

KITD816V mutations are often also detected in non-MCs (CD34+ hematopoietic progenitor cells, B-lymphocytes, monocytes, neutrophils, eosinophils, and occasionally T-lymphocytes), with variable patterns of involvement, indicating that SM is a disorder of a pluripotent hematopoietic progenitor cell [4]. Significantly more patients with AdvSM carry a KIT D816V+ multilineage involvement in their non-MCs myeloid compartment compared to patients with indolent forms [4,10]. 

The *KIT* median variant allele frequency (VAF) is strongly correlated with disease activity and burden as represented by serum tryptase level, disease subtype (indolent versus AdvSM) and survival, but not with the degree of MC infiltration of the BM [11]. The lack of correlation between the VAF and the degree of MC infiltration in the BM may be explained by the fact that non-MC cells in the BM also harbor a *KIT* mutation. Moreover, the presence of a high KITD816V VAF in the peripheral blood in patients without circulating MCs is highly predictive of an associated non-MC hematological neoplasm [11].

As with other myeloid neoplasms, additional mutations in genes encoding for epigenetic regulators (*ASXL1*, *EZH2*, *IDH2*, *TET2*), splicing factors (*SRSF2*, *SF3B1*, *U2AF1*), signaling molecules (*CBL*, *JAK2*, *N/KRAS*), or transcription factors (*RUNX1*) have been reported in SM with significantly higher mutation frequencies in AdvSM versus more indolent forms [12]. Sixty percent of patients harbor two or more mutations, in addition to KITD816V. The currently favored mechanistic concept of AdvSM pathogenesis is of a multimutated neoplasm, in which mutations in *TET2*, *SRSF2* and/or *ASXL1* in a pluripotent hematopoietic precursor cell might precede the KITD816V mutation. The latter molecular alteration represents a “phenotype modifier” of clonal hematopoietic stem cell disorders for SM [12].

In the last 10 years, the role of the additional mutations other than KITD816V has been investigated also regarding their impact on overall survival (OS) and response rates to disease-modifying approaches. A cluster of mutations, such as *SRSF2*, *ASXL1,* and *RUNX1*, was defined as an “S/A/R” complex. It was found to impact negatively on prognosis independently of conventional treatment and target therapies [12,13]. 

The Mayo Alliance Prognostic System (MAPS) for SM developed a prognostic score in which the presence of adverse mutations (*ASXL1/RUNX1/NRAS*) was recognized as an independent risk factor [14]. Similarly, the German registry-derived mutation-adjusted risk score for AdvSM (MARS) associated a worse OS with the number of concurrent mutations in the panel *SRSF2/ASXL1/RUNX1* [15]. The MARS system was independent of the WHO classification type and was confirmed in an independent validation set [16]. Recently, attention was given to the *SETD2* gene due to its implication in cancer. The human *SETD2* gene is located on chromosome 3 and is frequently targeted by copy number loss in various types of neoplasm. The SETD2 protein is involved in transcriptional activation and DNA repair. Martinelli et al. reported a loss of function mutations of *SETD2* in 53 SM patients and suggested that reduced SETD2 expression/absence might potentiate the effects of KIT constitutive activation to determine the phenotype of AdvSM [17].

## 3. Clinical Presentation and Diagnostic Subtypes

When a diagnosis of SM is made on the basis of the aforementioned major and minor criteria, it can be classified into five variants: indolent SM (ISM), smoldering SM (SSM), SM with an associated hematological (non-MC lineage) neoplasm (SM-AHN), aggressive SM (ASM) and mast cell leukemia (MCL) [1,2].

To discriminate between these different subtypes the diagnostic assessment should investigate signs of disease burden and organ damage, respectively, called ‘B’ and ‘C’ findings [1,2]. There are three types of ‘B’ findings: MC infiltration > 30% on BM biopsy and serum total tryptase > 200 ng/mL; signs of dysplasia or myeloproliferation in non-MC lineages; organomegaly (hepatomegaly, splenomegaly, lympho-adenopathy) with no evidence of organ function impairment [1,2]. 

The ‘C’ findings are single or multi-lineage cytopenia (absolute neutrophil count < 1.0 × 10^9^/L, hemoglobin < 10 g/dL, platelet count < 100 × 10^9^/L); palpable splenomegaly with hypersplenism; hepatomegaly with impairment of liver function, ascites and/or portal hypertension; skeletal involvement with large osteolytic lesions with/without pathological fractures; malabsorption with weight loss due to gastrointestinal MC infiltration [1,2]. 

Almost one ‘C’ finding is necessary for ASM diagnosis; ISM is defined if <2 ‘B’ findings are fulfilled and SSM if two or more ‘B’ findings are identified. Both ISM and SSM do not encompass ‘C’ findings [1,2]. 

Data collected from the largest series on SM patients in the pre-tyrosine kinase inhibitors (TKIs) era highlighted that OS varies across disease phenotypes, ranging from an unreached median OS for ISM to a 41-, 24- and 2-month median OS for ASM, SM-AHN, and MCL, respectively [18]. 

ISM is the most common variant of SM (~50%) and is characterized by a slowly progressive clinical course with a life expectancy comparable to the general population. In contrast to other SM categories, ISM consists of a low MC burden disease, predominantly characterized by MC mediator release symptoms, frequently with skin involvement. Urticaria pigmentosa-like skin lesions have a high prevalence reported between 66% and 75% [1,2].

Compared to ISM, SSM was significantly associated with older age and, as expected from disease definition, with higher bone marrow mast cell burden, higher serum tryptase level, and higher prevalence of palpable hepato-splenomegaly. SSM, as opposed to ISM, was also associated with adverse disease features including anemia, thrombocytopenia, and higher serum alkaline phosphatase level; in contrast, there was no significant difference in the expression of MC mediator symptoms, abnormal karyotype or KIT mutational frequency. Survival appeared significantly shorter in SSM vs. ISM (HR 5.5, 95% CI 2.8–10.2). However, the significant difference in survival between ISM and SSM was not sustained during age-adjusted multivariable analysis [1,2,19]. 

AdvSM is a term used to refer not only to ASM but also to SM-AHN and MCL. 

ASM has ≥1 C-findings indicating organ damage by infiltration of MCs and it was the third most common subgroup of SM. Patients with ASM frequently display constitutional symptoms (60%), hepatosplenomegaly (50%), and lymphadenopathy (30%). Severe anemia or thrombocytopenia interest about 30% of this patient population. Generally, serum tryptase has markedly elevated levels [2]. In the Mayo Clinic cohort, the overall median survival was 41 months and leukemic transformation occurred in two patients (5%) [2,18]. In the European Conference Network on Mastocytosis (ECNM) registry data, the median OS for 62 ASM patients was 5.7 years (10-year OS 44%) [16].

MCL is a rare and aggressive form of SM seen in <5% of SM patients and it is rapidly fatal with a median survival of 2–31 months. It is defined as SM with MCs ≥ 20% of marrow cells in a BM aspirate and/or ≥10% of total white blood cells in the peripheral blood, which are usually atypical, immature and may include metachromatic blasts. Skin lesions are usually absent, while MC mediator-related symptoms are frequently identified. MCL can be distinguished into de novo/primary MCL and secondary MCL when an anteceding MC neoplasm is present. The vast majority of secondary MCLs evolve from SM-AHN (~80%) and a minority from ASM, while a direct evolution of MCL from ISM is rare [1,2,18]. MCL can be further subdivided into chronic versus acute MCL and leukemic versus aleukemic MCL. Acute MCL is the most frequent and has a more aggressive course; the distinction between chronic versus acute MCL is based on the presence of C-findings and chronic MCL has no C-findings, whereas acute MCL has ≥1 C-finding [20]. 

Leukemic MCL is characterized by at least 10% of blood leukocytes being MCs. A KIT D816V mutation is found in only 46–68% of all MCL cases, being lower than in other advanced SM subtypes.

Commonly observed mutated genes in MCL are *TET2*, *SRSF2*, *ASXL1*, and *K/N-RAS*. At least one of the latter three mutations is found in 52% of patients with MCL [21].

SM-AHN is defined by the concomitant evidence of SM and another myeloid or lymphoid neoplasm independently meeting the WHO’s criteria [1,2]. Myeloid neoplasms can be identified in 80–90% of the cases, mostly represented by myeloproliferative neoplasm, myeloproliferative/myelodysplastic syndrome, chronic myelomonocytic leukemia, or acute myeloid leukemia. Rare cases of SM have been associated with lymphoma, chronic lymphocytic leukemia or plasma cell disorders [2,5]. The SM component may be either ISM, SSM, ASM or MCL. In the majority of patients (~70%) AHN is diagnosed concomitantly with SM, but there might be a long interval (3–370 months) between the two diagnoses [2,5]. Overall median survival in SM-AHN is 24–85 months [2,5]. KIT mutations are the most frequent alteration in SM-AHN, found in about 85% of patients, generally with a multilineage involvement. Additional non-KIT mutations are frequently identified in patients with SM-AHN and mainly consist of mutations in *TET2*, *SRSF2*, *ASXL1*, *RUNX1*, *N/KRAS*, or *IDH2* [21]. Karyotypic abnormalities are detected in 19–32% of patients with SM-AHN, mostly deletions (del [5q], del [1q], del [12q], less frequently del [7q]), followed by trisomies (+8), monosomies (–7), and complex karyotypes [22]. Leukemic transformation (~10–15% overall) was seen significantly more frequently in SM with myelodysplastic syndrome [2,18]. 

## 4. Treatment of Systemic Mastocytosis

Clinical presentation of ISM is generally dominated by skin involvement (urticaria pigmentosa-like skin lesions) and MC mediator-related symptoms (such as flushing, pruritus, anaphylactoid reactions, gastrointestinal symptoms, osteopenia or frank osteoporosis). Conversely, in AdvSM, the predominant clinical problem is usually not related to mediator-associated symptoms, but to the proliferation and often aggressive growth of MCs leading to organomegaly and even organ failure [1,2].

The goal of ISM treatment is to ameliorate and prevent mediator-related symptoms, as only a neglectable fraction of patients will progress to higher grade disease and OS is quite superimposable to the general population. The advanced forms may require cytoreductive therapy to reverse end-organ damage caused by MC infiltration [2]. Until the first decade of the 21st century, the mostly used cytoreductive drugs were represented by hydroxycarbamide, interferon-alpha (INF-α), and 2-chlorodeoxyadenosine (cladribine or 2-CdA) [23]. With the advent of the TKI era, many efforts have been made to find a proper inhibitor of SM *KIT* driver mutation, and midostaurin was the first TKI approved for the treatment of AdvSM which has been shown to induce major clinical responses [24].

### 4.1. Current Treatment Approaches for Mast Cell Mediator-Related Symptoms

Treatment approaches in ISM and SSM patients should be tailored on personal clinical history, type and severity of mediator-related symptoms [2,25].

ECNM guidelines recommend that every SM patient, regardless of the presence of mediator symptoms, history of anaphylaxis, or bone disease, should be preferentially managed and followed by a multidisciplinary team with at least a hematologist, an allergologist and an endocrinologist, should assume adequate intake of calcium and vitamin D, and undergo periodical radiological evaluation (spine X-ray and bone marrow density). Training on self-administration of epinephrine and antihistamine drugs in case of warning symptoms preceding an anaphylactic reaction is paramount and avoiding known potential triggers for anaphylaxis should be pursued, particularly in the perioperative setting [26]. 

Skin symptoms such as flushing, itching, angioedema and dermatographism should be approached in a stepwise manner with frontline H1-antihistamine and in case of refractoriness by adding a leukotriene receptor antagonist (e.g., montelukast) or sodium cromoglycate, which acts as MC stabilizer and inhibits MC degranulation [26]. 

Likewise, gastrointestinal symptoms (abdominal pain, cramping, nausea, vomiting and diarrhea) should be managed sequentially with up-front H2-antihistamines (e.g., ranitidine, famotidine). A proton pump inhibitor should be considered in case of inefficacy; while sodium cromoglycate should be administered as a third-line treatment [26]. 

A newly emerging area of interest concerns passive immunotherapy targeting IgE-dependent MC degranulation since several retrospective cohort studies and case series suggested the potential clinical application in SM patients, particularly those complaining of recurrent episodes of anaphylaxis [27,28,29,30]. Omalizumab is a humanized IgG1 monoclonal anti-IgE antibody, approved for moderate-to-severe asthma, steroid-refractory nasal polyps and chronic spontaneous urticaria [31]. 

Targeting IgE binding site to FcƐRI receptors on MCs and basophils surfaces, omalizumab leads to inhibition of IgE-mediated MC activation by preventing IgE-allergen complexes deposition on immunoglobulin receptors. Recently, a systematic review including 16 published reports and retrospective studies on omalizumab in ISM patients resulted in amelioration of cardiovascular, gastrointestinal, and cutaneous mediator-related symptoms, and in complete symptom resolution in 43%, 29% and 27% of patients, respectively, with a rapid onset of clinical response with a mean time to first response of 2.3 months. Among all patients, 84% of those who experienced multiple episodes of severe idiopathic anaphylaxis showed complete resolutions of symptoms [32]. 

A prospective, double-blind, randomized placebo-controlled multicenter trial explored the clinical effects measured as a specific symptom-score point-reduction from baseline through a treatment course of 6 months. Of the 17 patients enrolled, only 14 received medications (6 administered with omalizumab and 8 with placebo). Although the absolute decrease in the severity of symptoms was more pronounced in the experimental arm, statistical significance was not reached. The safety profile of the experimental arm was acceptable with no difference in adverse events between the two arms. The most frequent adverse reactions occurring in patients receiving omalizumab were dizziness, infusion-related reaction and muscle pain [33]. 

### 4.2. Future Potential Therapeutic Targets for Mast Cell Mediator-Related Symptoms

MCs activity relies upon different mediators which can be categorized into three subclasses: (a) granule stored preformed mediators (histamine, proteoglycans, MCs specific proteases (e.g., chymase, tryptase, carboxypeptidase-3)); (b) de novo synthesis lipidic mediators (prostaglandin E2, D2; leukotriene B4, C4); (c) cytokines (e.g., TNF-α, TGF-β, IL-1, IL-4, IL-5) [5,6]. 

A review by Caughey et al. concluded that MCs proteases (most of all chymase and tryptase) could be used as a scaffold for future development of protease inhibitor drugs in MC diseases [34].

Chymase is a chymotrypsin-like enzyme released as a preformed mediator upon MC degranulation and displays an extensive activity spectrum on several extracellular matrix molecules (fibronectin, laminin, matrix metalloproteases), cytokines (IL-1, IL-6, IL-8) and vasoactive peptides (angiotensin I; endothelin-1) [35]. The homeostatic effects of chymases and their experimental inhibitors have been largely explored in murine models and the current literature proposes that they could exert either a protective or detrimental effect on cardiovascular aspects (such as pulmonary hypertension or dysmetabolic diseases) [36,37].

Tryptase is the most abundant prestored secretory MC protease, and it is the most reliable marker for MCs at any stage of maturation, although it is non-MC restricted. Several substrates of tryptase enzymatic activity have been identified and tissue effects can be proinflammatory, angiogenic and reparative [38]. So far, tryptase activity has been demonstrated to play an in vivo crucial role in various inflammatory diseases and cancers [39,40,41]. The most represented isoform is β-tryptase, which is a “self-assembling protease” as its inactive monomeric isoform undergoes intragranular processing, consisting of the self-removal of a signal peptide by its endopeptidase activity and subsequent polymerization into heparin and glycosaminoglycan-stabilized tetramers, which constitutes the biologically active counterpart [38]. 

Accumulating data on tryptase molecular structure and conformational modifications paved the way for the development of a humanized anti-tryptase antibody (MTPS9579A) which demonstrated activity in both in vitro and in vivo preclinical models of severe type II asthma reducing IgE-dependent MC degranulation by dissociating the active tetramer into inactive monomers [42]. Recently, a first phase I human randomized placebo-controlled trial in healthy subjects receiving MTPS9579A at different dose levels showed a good safety profile and provided the proof of concept of its pharmacodynamical activity as it reduced tryptase tetramers both in nasal fluid and serum [43]. 

A target protein being explored is represented by KIT which represents a master regulator of MC biology. A KIT-targeting antibody has been shown to efficiently deplete MCs in mice and recently the anti-KIT monoclonal antibody CDX-0159 was evaluated in a placebo-controlled phase 1a healthy volunteer. CDX-0159 was demonstrated as a potent inhibitor of KIT signaling and MC activation. Moreover, CDX-0159 induced a dose-dependent, profound suppression of plasma tryptase, indicative of systemic MC suppression or ablation [44].

### 4.3. Interferon-α (INF-α)

Since 1992, the administration of (IFN)-α has demonstrated potential benefits in the treatment of mastocytosis. INF-α can decrease symptoms of MCs degranulation and BM MC infiltration and can improve hepatosplenomegaly, and, in particular, bone density. However, the overall response rate (ORR) is approximately 20%, and no fixed dose or duration has been established. IFN-α treatment can be complicated in up to 50% of patients by toxicities, including flu-like symptoms, bone pain, fever, cytopenias, depression, and hypothyroidism with a consequently high dropout rate [18,45,46].

To ameliorate tolerability and reduce discontinuation, INF-α has been administered in combination with prednisone. In the Mayo Clinic study, 47 SM patients received INF-α with or without prednisone. The dosage of INF-α ranged from 3.5 million units (MU) to 30 MU per week with an initial dosage of prednisone ranging from 20 mg to 60 mg per day. The ORR of the 40 evaluable cases was 53% (ISM and ASM 60%; SM-AHN 45%); a complete remission (CR) was observed in only 3% of the patients. The overall median duration of response was 12 months, and the responses were not significantly different between the arms with or without prednisone [18].

In a French study, 20 SM patients (16 ASM and 4 ISM) were treated with IFN-α starting at 1 MU/day with a progressive increase to 5 MU/m^2^/day. All 13 patients who were treated for at least 6 months exhibited responses in circulating MC mediator levels, but not in a BM MC burden. Adverse effects were frequent and four responding patients experienced a prompt relapse of symptoms after treatment cessation [46].

### 4.4. Chlorodeoxyadenosine (Cladribine or 2-CdA)

Conventional chemotherapy with chlorodeoxyadenosine (cladribine or 2-CdA) is usually administered to SM patients with a high tumor burden and rapidly progressive disease, achieving an ORR ranging from 50% to 72% [18,47,48]. 

In the Mayo Clinic study, 26 patients were treated intravenously with a dose of 5 mg/m^2^ per day or 0.13–0.17 mg/kg per day for 5 days. The median number of cycles was three (range, 1–9). The ORR of the 22 evaluable patients was 55% (56% for ISM and 50% for ASM). The median duration of response was 11 months and the main toxicities were infection linked to myelosuppression [47]. 

In a French study, 68 adult SM patients intravenously or subcutaneously received cladribine 0.14 mg/kg for 5 days every 4–12 weeks up to nine cycles. The median number of cycles administered was 3.7 and ORR was 72% (92% for ISM and 50% for AdvSM). After a median follow-up of >10 years, the median duration of response was 3.71 and 2.47 years for indolent and aggressive SM. Immunosuppression and opportunistic infections represented the most frequent grade 3 or 4 toxicities as the main toxicities, and the latter occurred as grade 3 or 4 toxicities in a minority of patients (13%) [48].

### 4.5. Tyrosine-Kinase Inhibitors

#### 4.5.1. Imatinib Mesylate

Imatinib mesylate (STI571) is an orally bioavailable multikinase inhibitor approved for clinical use in chronic myeloid leukemia, Philadelphia-positive acute lymphoblastic leukemia, CD117+ gastrointestinal stromal tumors, and also for myeloid/lymphoid neoplasms with eosinophilia and *PDGFR* rearrangements [49]. 

Although *KIT*-wild type receptor is encompassed in the clinical spectrum of imatinib targets, data on in vitro and in vivo efficacy on *KIT* mutated SM showed contrasting results [50,51,52,53,54,55]. Some rare types of *KIT* extracellular and juxtamembrane domain mutants, as well as its wild type isoform, have been proven to be imatinib-sensitive in vitro; conversely, the most common KITD816V kinase domain mutant is not inhibited by imatinib [50,51,52,53,54,55]. 

A phase II prospective trial evaluated the efficacy and safety of imatinib at a dosage of 400 mg daily in 14 patients with SM (12 ISM/SSM, 2 ASM) [52]. Eleven patients (78%) carried a KITD816V mutation. The response criteria were defined considering: reduction in tryptase level, BM MCs infiltration and reduction in organomegaly and symptoms. CR was defined in case of absence of symptoms, <5% MCs in BM aspirate, no focal infiltrates of MCs, complete disappearance of skin lesions and normalization of serum tryptase levels. The major response was defined as a reduction of >50% in serum tryptase levels or skin lesions, and <10% MCs in BM aspirate. In the entire study population, a CR was attained in one patient and five major responses were achieved among patients with ISM. The only patient with ASM and KITD816V mutation did not respond [52]. 

Two retrospective experiences, one from an Italian multicenter trial and the other from Mayo Clinic [23,53], demonstrated a rate of the overall response of 29% and 18%, respectively. Subgroup analysis for KITD816V mutated patients confirmed no response in *KIT*-positive patients among the Italian cohort [53]. The ORR was 17% for *KIT*-positive patients and 33% for *KIT*-negative patients in the Mayo Clinic cohort [23].

Results from a phase II study from M.D. Anderson Cancer Center conducted on 20 SM patients (4 ASM, 5 SM-AHN and 11 ISM) showed an improvement in SM-related symptoms in ISM regardless of KITD816V mutation. Only one patient with SM associated with hypereosinophilia syndrome achieved a CR. No clinical benefit was observed in patients with an aggressive disease [54].

In all these studies, imatinib was well tolerated with non-hematologic G3–4 adverse events occurring in one-third of the cases. Hematologic G3–4 toxicity interested nearly 20% of the patients, with resolution after temporary drug discontinuation [50,51,52,53,54,55]. 

On this basis, imatinib was approved by the U.S. Food and Drug Administration (FDA), but not by the European Medicines Agency (EMA), in 2006 for ASM patients without D816V mutation or unknown *KIT* mutation status. 

More recently, a clinical trial carried out by the Spanish group showed a response to imatinib in 5/10 patients with SM lacking exon 17 *KIT* mutations, which included three patients with the K509I *KIT* mutation, one patient with wild-type *KIT* SM-chronic eosinophilic leukemia who had no *PDGFR* rearrangements and one patient with wild-type *KIT* SM [56]. 

Together with data from a critical review of all published cases of SM treated with imatinib, these observations support that response to imatinib relies on the presence of imatinib-sensitive mutations either involving *KIT* (e.g., juxtamembrane or transmembrane *KIT* mutations) or *PDGFR* (e.g., *FIP1L1/PDGFRA* rearrangement) rather than on the absence of the D816V *KIT* mutation [50,51,52,53,54,55,56].

#### 4.5.2. Masitinib

Beyond imatinib, both preclinical and clinical trials investigated the role of other TKIs. Specifically, masitinib is an orally bioavailable TKI that showed in vitro activity against PDGFR, Lyn, Fyn, and wild-type KIT [57]. Clinical studies of masitinib in mastocytosis patients were mainly focused on exploring its potential utility for MC-mediator-associated symptoms [58,59]. 

In a phase II trial, 25 patients with cutaneous mastocytosis or SM with disabilities associated with mediator-associated symptoms were treated with an initial dose level of 3 or 6 mg/kg/day over 12 weeks. Overall clinical response was achieved in 56% of patients with sustainable improvement observed throughout the extension phase (>60 weeks). More frequent adverse events were edema (44%) and nausea (44%) [58]. 

In phase III randomized, placebo-controlled study, masitinib was administered at 6 mg/kg per day in two daily doses for 24 weeks in ISM and cutaneous mastocytosis patients. Masitinib demonstrated only modest efficacy in symptoms control, which occurred at 24 weeks in 18.7% and 7.4% for masitinib and placebo control arm, respectively. The safety profile of masitinib was acceptable, with no life-threatening side effects; most adverse events occurred during the first 6 months and were generally non-hematologic (nausea/vomiting) and cutaneous, leading to dose discontinuation in only a few cases (1.5%) [59].

#### 4.5.3. Midostaurin

Midostaurin (PCK412) is an orally bioavailable multikinase inhibitor, approved by the U.S. FDA and EMA for the treatment of adults with ASM, SM-AHN and MCL. Moreover, midostaurin is now approved also for FMS-like tyrosine kinase 3 (*FLT3*) mutated acute myeloid leukemia [60]. 

Midostaurin competitively binds to the ATP binding site in the catalytic domain of tyrosine kinases, resulting in their inhibition. Besides its activity against *FLT3*, midostaurin was demonstrated to inhibit both wild-type and D816V-mutated *KIT*, as well as additional protein kinases such as kinase insert domain-containing receptor (*KDR*), fibroblast growth factor receptor (*FGFR*), vascular endothelial growth factor receptor 2 (*VEGFR2*), *FIP1L1/PDGFRa* fusion protein, and members of the serine/threonine protein kinase C (*PKC*) family [61]. 

The first clinical data come from a phase II multicenter trial in which oral midostaurin was administered at 100 mg twice daily over 28-day cycles in 26 patients with AdvSM [62]. 

Three ASM, seventeen SM-AHN, and six MCL patients with at least one sign of organ damage were enrolled in this trial. Data were encouraging both for efficacy and safety. ORR was 69% and clinical benefits were observed in all AdvSM variants. The most frequent grade 3/4 non-hematologic and hematologic toxicities were asymptomatic and represented by hyperlipasemia (15%) and anemia (12%). Midostaurin produced a ≥50% reduction in BM MC burden and serum tryptase level in 68% and 46% of patients, respectively. Median OS for the entire cohort was 40 months, and 18.5 months for MCL patients. Low-grade gastrointestinal side effects were common and manageable. With a median follow-up of 10 years, no unexpected toxicities emerged [62].

On the basis of the aforementioned reports, a multicenter international single-arm phase II study was designed. Midostaurin (100 mg twice a day) was continuously administered both in first-line and in relapsed/refractory settings in 116 patients with AdvSM, of whom 89 patients were evaluable for response [24]. 

The ORR was 60% in the global efficacy population with major and partial responses of 45% and 16%, respectively. ORR for ASM, SM-AHN and MCL subgroups were 75%, 58% and 50%, respectively. A difference in response rate was not observed between treatment-naive patients and patients who received prior therapies (62% vs. 58%, respectively). This pivotal trial confirmed the role of midostaurin in reducing disease burden: a reduction of ≥50% was observed from the baseline for serum tryptase levels, BM MCs burden and spleen volume in 60%, 58% and 26% of patients, respectively. Response rates were independent of the presence of KIT-D816V mutation; indeed, the ORR ranged from 44% to 75% and from 40% to 70% among three major disease subgroups in *KIT*-positive and *KIT*-negative patients. Median OS and progression-free survival (PFS) were 28 and 14 months. Factors negatively affecting OS were MCL subtype, non-responders and exposure to prior therapy. The safety profile was more than acceptable; the most frequent adverse events were low-grade nausea, vomiting, and diarrhea. New or worsening grade 3 or 4 neutropenia, anemia, and thrombocytopenia occurred in 24%, 41%, and 29% of the patients, respectively, mostly in those with pre-existing cytopenias. Dose reduction owing to toxic effects occurred in 56% of the patients; re-escalation to the starting dose was feasible in 32% of those patients [24]. 

In 2016, the French group published data from a prospective survey about patients with SM who were treated with midostaurin under a transitory-use authorization program [63]. Twenty-eight patients received midostaurin (including 4 ASM, 18 SM-AHN, and 3 MCL); these patients were compared with a control group treated with different drugs. For the midostaurin cohort, the ORR was 71%; after a mean follow-up of about 18 months, OS was 42.7% for patients treated with midostaurin and 14.9% for the control group, testifying to the single-agent activity of midostaurin in AdvSM. Digestive discomfort was confirmed as the most frequent side effect [63].

More recently, a German study compared the outcome of patients included in the pivotal trial of midostaurin and a historical cohort of 46 patients treated with therapies other than midostaurin, revealed a two-fold increase in OS in the group of patients treated with midostaurin (41.4 vs. 19.5 months) [64]. 

On the basis of data from the German Registry on Disorders of Eosinophils and Mast Cells, data about the efficacy of midostaurin and cladribine in patients with AdvSM were compared.

Sixty-three patients treated with midostaurin were considered together with 23 patients treated with cladribine and patients who received sequentially midostaurin-cladribine (n. 30) or cladribine-midostaurin (n. 23). Midostaurin monotherapy was superior to cladribine monotherapy with significant longer OS (median 4.2 vs. 1.9 years) and leukemia-free survival (2.7 vs. 1.3 years). The use of midostaurin in any line compensated for the inferior efficacy of cladribine. The combination of MARS score and the reduction of KITD816V allele burden at month 6 allowed us to distinguish three risk categories with a significantly different OS [65]. 

The impact of clonal architecture on responsiveness to midostaurin was explored in a group of 38 AdvSM patients [16]. This study confirmed the negative prognostic impact of additional molecular aberrations in *SRSF2*, *ASLX1*, or *RUNX1*. ORR was significantly different between S/A/R negative (75%) and S/A/R positive (39%), in the same way, the median OS was 27 months for S/A/R positive patients and not reached for S/A/R negative patients [13].

Notably, a comparison between a historical cohort treated in the pre-TKI setting and S/A/R positive AdvSM treated with midostaurin showed a significant difference in median OS (14 months vs. 40 months), validating midostaurin efficacy also in an adverse genetical subgroup [13]. 

The capacity of midostaurin to improve mastocytosis-related symptoms in patients with AdvSM led to exploring its potential utility also in severely symptomatic non-AdvSM refractory to conventional anti-mediator therapies.

A single-center phase II trial was conducted on 20 ISM patients with severe mediator-related symptoms [66]. Results showed a 35% and 38% reduction in the severity of symptoms when assessed at 12 and 24 months, respectively; a similar improvement in quality of life at 24 months was achieved. The majority of patients who discontinued midostaurin at the end of treatment (24 months) experienced a recrudescence in mediator-related symptoms [66]. 

A retrospective series on midostaurin use in ISM and SSM patients included in the Mayo clinic cohort was recently published [67]. Thirteen patients were recruited; in the 19-month median times from midostaurin initiation and last follow-up, 62% and 44% of the patients had improvement in MC-related mediator symptoms and MC skin lesions, respectively. Moreover, clinical responses were associated with a decrease of MCs BM in 4 out of 5 BM-evaluable patients and serum tryptase in 9 out of 11 evaluable patients [67].

This study confirmed that midostaurin treatment could lead to a clinical benefit in ISM/SSM patients, but the tolerability was limited by a high incidence of gastrointestinal adverse events which needed dose reduction (nine patients) and/or treatment discontinuation (five patients). These observations raise questions about the use of midostaurin in a long-term period for an indolent disease [67].

#### 4.5.4. Avapritinib/BLU-285 

Avapritinib (BLU-285) is an oral selective *KIT* inhibitor with a high affinity for D816V mutant-*KIT* that was FDA-approved in June 2021 for adult patients with AdvSM.

The first evidence of avapritinib efficacy derived from in vitro studies in mouse models of systemic disease and highlighted that BLU-285 has a broad spectrum of activity against ATP-binding activation loop mutants (D816V, D8816Y, V560G), is resistant to imatinib treatment and V654A, N655K and D677N mutants and is generally resistant to midostaurin treatment [68]. 

EXPLORER-1 was a phase I dose-finding clinical trial which investigated avapritinib in patients with AdvSM [69]. Of the total 86 enrolled patients, 69 had a confirmed AdvSM and 53 were evaluable for response. More than 90% of patients were KIT-D816V- or D816Y-positive and 70% of them had a diagnosis of SM-AHN. Nearly 60% of patients received a prior line of systemic therapy (including midostaurin) and 50% were S/A/R positive [69]. 

ORR was 75% and the median duration of response was 38 months. Substantial differences were observed between midostaurin naive (ORR 83%) and midostaurin-exposed patients (ORR 59%). Notably, response rates between S/A/R positive (ORR 77%) and S/A/R negative (ORR 74%) cases did not significantly differ. At a median follow-up of 23 months, 14 patients (20%) experienced a clinical progression. The estimated 24-month OS was 76% and it was homogeneous across all disease subtypes with a rate of 100%, 67% and 92% for patients with ASM, SM-AHN and MCL, respectively. 

Treatment with avapritinib was generally well-tolerated. The most common adverse reactions (incidence ≥ 20%) were edema, diarrhea, nausea, and fatigue/asthenia [69].

Nine cases of intracranial bleeding were documented, four with a subdural hematoma and five with intracranial hemorrhage. Notably, seven of these events were associated with antecedent grade ≥ 3 thrombocytopenia. On this basis, avapritinib is now not recommended for the treatment of patients with AdvSM with platelet counts of less than 50 × 10^9^/L [69].

The phase II study PATHFINDER is still ongoing; a preliminary interim analysis of 32 enrolled AdvSM with measurable ‘C’ findings who had sufficient follow-up (median of 10.4 months) supported the results derived from the previous phase I study [70].

The ORR was 75%, including 19% with complete remission with full or partial hematologic recovery. Reductions of ≥50% from baseline in serum tryptase (93%), BM MCs (88%) and KITD816V variant allele fraction (60%) were observed. The most frequent grade ≥ 3 adverse events were neutropenia (24%), thrombocytopenia (16%) and anemia (16%). Only one intracranial bleeding was registered in one patient with G3 thrombocytopenia [70]. 

Beyond activity of the MC lineage, avapritinib treatment also led to a reduction of the associated myeloid neoplasm burden on peripheral blood. A reduction of ≥50% of monocytes and eosinophils peripheral counts from baseline in near 80% of patients with SM plus chronic myelomonocytic leukemia or SM and associated eosinophilia/chronic eosinophilic leukemia were observed [70].

The ongoing PIONEER study is a multicenter, randomized phase II trial that is investigating the safety and efficacy of avapritinib in patients with ISM with moderate-severe symptoms [71]. Preliminary results showed that safety at the recommended dosage of 25 mg was comparable with placebo with no grade 3 toxicities or dose reduction needed. The most common grade adverse events across all dose groups were nausea (37%), dizziness (33%), headache (30%) and diarrhea (23%). Moreover, no patients experienced grade 4–5 adverse events and treatment-related hematologic toxicities were not documented in the 25 mg cohort. As efficacy data are pending, a significant mean reduction in total symptom score was observed across all avapritinib cohorts at 16 weeks of treatment when compared to placebo with a 30% vs. 3% mean reduction from baseline. Furthermore, the reduction of KITD816 variant allele fraction, serum tryptase and BM MCs were statistically superior in the experimental arm when compared to the placebo cohort [71]. 

A summary of published trials of avapritinib and other TKIs target therapies for SM patients is reported in Table 1.

#### 4.5.5. Other Investigational Tyrosine-Kinase Inhibitors

Ripretinib (DCC-2618) is a novel type II tyrosine switch control inhibitor for the treatment of KIT- and/or platelet-derived growth factor receptor A (PDGFRA)-driven cancers, including gastrointestinal stromal tumor (GIST). In May 2020, oral ripretinib received its first approval in the USA for the treatment of adult patients with advanced GIST who have received prior treatment with ≥3 kinase inhibitors, including imatinib [72]. The safety and tolerability of DCC-2618 in patients with advanced malignancies, including SM, is under study (NCT02571036). 

BLU-263 is an investigational, potent, and selective oral small-molecule inhibitor of KIT which was designed to inhibit KITD816V with minimal central nervous system penetration as compared to avapritinib. The safety and tolerability of BLU-263 were documented in phase 1, a randomized, double-blinded, placebo-controlled study in healthy volunteers [73]. BLU-263 is under evaluation in a randomized, double-blind, and placebo-controlled phase 2/3 study in patients with ISM whose symptoms are not adequately controlled by the best supportive care (NCT04910685). 

Bezuclastinib (CGT9486) is an oral, highly selective TKI with potent activity against KITD816V. It was designed to avoid other closely related kinases and minimal brain penetration has been observed with bezuclastinib. Bezuclastinib has shown preliminary clinical activity and a tolerable safety profile in patients with advanced solid tumors including GIST [74]. A phase 2 open-label multicenter clinical study of the safety and efficacy profiles of CGT9486 as a single agent in patients with AdvSM is ongoing (NCT04996875). 

### 4.6. The Role of Allogeneic Stem Cell Transplantation in the Age of KIT Inhibitors

The role of allogeneic hematopoietic stem cell transplantation (all-HSCT) in AdvSM is a field of discussion due to the low numbers of patients who underwent transplantation and lacking prospective trials [2,75,76]. 

The largest retrospective study involves 57 patients with SM (38 SM-AHN; 7 ASM; 12 MCL) [75]. Forty patients (70%) achieved a response at day +100. All cases of SM-AHN achieved a response, while up to half of MCL patients were primarily refractory. Three-year OS was 74% for patients with SM-AHN, 43% for ASM, and 17% for MCL. The use of reduced-intensity conditioning and progressive disease were identified as adverse prognostic factors [75]. 

Even though not matched for baseline characteristics, the comparison between the transplantation experience and the cohort treated with midostaurin highlights that 3-year OS for midostaurin patients was relatively better for ASM (65%) and MCL (26%), but lower for SM-AHN (44%) [24]. Regarding the avapritinib experience, 2-year OS survival rates were 70%, 100%, and 88% for SM-AHN, ASM, and MCL patients, respectively [69]. These data suggest that among AdvSM subtypes, SM-AHN patients may be selected for consideration of allo-HSCT. The AHN variant and disease status should play a crucial role in the final decision. Moreover, ASM patients with relapsed/refractory disease and those with acute MCL might be considered as a possible eligible population for allo-HSCT, especially if younger and healthier patients have a suitable fully HLA-matched donor [76]. 

In brief, the decision to use allo-HSCT in AdvSM, as in other hematological malignancies, should be multifactorial and must consider patient, donor, and disease characteristics. 

## 5. Conclusions

SM can be extremely heterogeneous and its treatment should be highly individualized; while the backbone of therapy in ISM and SSM is symptom management, for aggressive and leukemic forms, cytoreductive treatment is indicated. The SM pathogenesis driven by KIT mutations led to exploring the potential utility of TKIs, but the evidence of resistance to the first-generation TKI imatinib in D816V-positive cases induced the development of new highly selective TKIs.

Midostaurin and avapritinib have demonstrated significant clinical activity regardless of KITD816V mutation. The improvement of molecular knowledge, such as the identification of the S/A/R profile and the emergence of new targeted therapies, changed the landscape of this disease, especially for advanced forms of SM. Precision medicine and personalized medicine approaches will improve management and the quality of life in patients with mast cell neoplasms. 

## Figures and Tables

**Table 1:** Selected prospective clinical trials of target therapies with TKIs in systemic mastocytosis.

Drugs	Type of Study	Patients	Outcomes
Imatinib [52]	Phase II	12 ISM/SSM + 2 ASM	CR 7%, major response 36%
Imatinib [54]	Phase II	11 ISM + 4 ASM + 5 SM-AHN	CR 5%
Masitinib [58]	Phase II	25 cutaneous mastocytosis or SM with disabilities associated with mediator-related symptoms	ORR 56%
Masitinib [59]	Phase III, placebo controlled	135 ISM/cutaneous mastocytosis	ORR 18.7%
Midostaurin [62]	Phase II	3 ASM + 17 SM-AHN + 6 MCL	ORR 69%, median OS 40 months
Midostaurin [24]	Phase II	16 ASM + 57 SM-AHN + 16 MCL	ORR 60%, median OS 28.7 months
Midostaurin [66]	Phase II	20 ISM patients with severe mediator-related symptoms	35% and 38% reduction in severity of symptoms, at 12 and 24 months, respectively
Avapritinib [70]	Phase II	9 ASM + 43 SM-AHN + 10 MCL	ORR 75% in 32 response-evaluable patients (CR 19%)
Avapritinib [71]	Phase II, randomized, double-blind, placebo-controlled	204 ISM	Reduction in total symptoms score at 16 weeks 30%

## Data Availability

No new data were created or analyzed in this study. Data sharing is not applicable to this article.
